# Cigarette smoke alters the ability of human dendritic cells to promote anti-*Streptococcus pneumoniae* Th17 response

**DOI:** 10.1186/s12931-016-0408-6

**Published:** 2016-07-26

**Authors:** Olivier Le Rouzic, Bachirou Koné, Jerome Kluza, Philippe Marchetti, Florence Hennegrave, Cécile Olivier, Gwenola Kervoaze, Eva Vilain, Clémence Mordacq, Nicolas Just, Thierry Perez, Nathalie Bautin, Muriel Pichavant, Philippe Gosset

**Affiliations:** 1 Univ. Lille, U1019 – UMR 8204 – CIIL – Center for Infection and Immunity of Lille, F-59000 Lille, France; 2 CNRS, UMR 8204, F-59000 Lille, France; 3 Inserm, U1019, F-59000 Lille, France; 4 CHU Lille, Service de Pneumologie Immunologie et Allergologie, F-59000 Lille, France; 5 Institut Pasteur de Lille, F-59000 Lille, France; 6 Univ. Lille, UMR-S 1172 – JPArc – Centre de Recherche Jean-Pierre AUBERT Neurosciences et Cancer, F-59000 Lille, France; 7 Inserm, UMR-S 1172, F-59000 Lille, France; 8 CHU Lille, Service de Pédiatrie, F-59000 Lille, France; 9 CH Roubaix, Service de Pneumologie, F-59100 Roubaix, France; 10 LI3, CIIL - Institut Pasteur de Lille, F-59000 Lille, France

**Keywords:** Chronic obstructive pulmonary disease, Dendritic cells, Streptococcus pneumoniae, Smoking, Interleukin-1β, Interleukin-23, Th17 cytokines

## Abstract

**Background:**

Chronic obstructive pulmonary disease (COPD) is associated with chronic inflammation and impaired immune response to pathogens leading to bacteria-induced exacerbation of the disease. A defect in Th17 cytokines in response to *Streptococcus pneumoniae*, a bacteria associated with COPD exacerbations, has been recently reported. Dendritic cells (DC) are professional antigen presenting cells that drive T-cells differentiation and activation. In this study, we hypothesized that exposure to cigarette smoke, the main risk factor of COPD, might altered the pro-Th17 response to *S. pneumoniae* in COPD patients and human DC.

**Methods:**

Pro-Th1 and -Th17 cytokine production by peripheral blood mononuclear cells (PBMC) from COPD patients was analyzed and compared to those from smokers and non-smokers healthy subjects. The effect of cigarette smoke extract (CSE) was analyzed on human monocyte-derived DC (MDDC) from controls exposed or not to *S. pneumoniae*. Bacteria endocytosis, maturation of MDDC and secretion of cytokines were assessed by flow cytometry and ELISA, respectively. Implication of the oxidative stress was analyzed by addition of antioxidants and mitochondria inhibitors. In parallel, MDDC were cocultured with autologous T-cells to analyze the consequence on Th1 and Th17 cytokine production.

**Results:**

PBMC from COPD patients exhibited defective production of IL-1β, IL-6, IL-12 and IL-23 to *S. pneumoniae* compared to healthy subjects and smokers. CSE significantly reduced *S. pneumoniae*-induced MDDC maturation, secretion of pro-Th1 and -Th17 cytokines and activation of Th1 and Th17 T-cell responses. CSE exposure was also associated with sustained CXCL8 secretion, bacteria endocytosis and mitochondrial oxidative stress. Antioxidants did not reverse these effects. Inhibitors of mitochondrial electron transport chain partly reproduced inhibition of *S. pneumoniae*-induced MDDC maturation but had no effect on cytokine secretion and T cell activation.

**Conclusions:**

We observed a defective pro-Th1 and -Th17 response to bacteria in COPD patients. CSE exposure was associated with an inhibition of DC capacity to activate antigen specific T-cell response, an effect that seems to be not only related to oxidative stress. These results suggest that new therapeutics boosting this response in DC may be helpful to improve treatment of COPD exacerbations.

**Electronic supplementary material:**

The online version of this article (doi:10.1186/s12931-016-0408-6) contains supplementary material, which is available to authorized users.

## Background

Chronic obstructive pulmonary disease (COPD) is a leading cause of morbidity and mortality worldwide mainly due to cigarette smoke exposure [1]. Oxidative stress induced by cigarette smoke induces a chronic lung inflammation responsible for a non-reversible airflow limitation and an impaired immune lung defenses leading to airway bacterial infections [2, 3]. Most of these are due to *Streptococcus pneumoniae*, *Haemophilus influenzae* and *Moraxella catarrhalis* [4]. These infectious episodes are the major cause of acute exacerbations which have a strong impact on mortality and on disease-related costs [5]. Indeed, about 50 % of COPD patients developing a first severe exacerbation die within 4 years after this episode [6]. Although studies reported that the mucosal inflammation is increased during COPD disease, recent evidences demonstrated that the immune response to micro-organisms is altered [7].

Dendritic cells (DC) are professional antigen presenting cells (APC) linking innate and adaptive immune responses, that are crucial to build an effective anti-bacterial response [8]. DC drive antigen-specific T-cells differentiation and activation in response to pathogens by delivering 3 signals including antigen presentation, co-stimulatory molecule expression and immuno-modulatory cytokine production [9]. The characteristics of these signals determine the polarization of the T-cell response as well as those of non-conventional lymphocytes [10]. Both Th1, i.e., IFN-γ, and Th17, i.e., interleukin (IL)-17 and IL-22, cytokines are needed to control *S. pneumoniae* infection [11, 12]. The oxidative stress induced by cigarette smoke inhibits LPS-induced DC maturation [13] and production of interleukin-12 (IL-12) and IL-23 which are involved in Th1 and Th17 T-cell differentiation, respectively [14]. However, there are little data on cigarette smoke effects on live bacteria-induced DC maturation.

Previous studies have shown that IL-17-producing cells are more frequent in the airways of steady-state COPD patients [15]. Conversely, another study have reported lower IL-17 levels during exacerbation in severe COPD patients compared to healthy subjects and mild COPD patients [16]. These results are strengthened by another clinical study confirming lower IL-17 blood levels in COPD patients colonized in the airways by opportunistic pathogens [17]. We recently described an altered IL-17 response to infection by *S. pneumoniae* in in-vitro stimulated peripheral blood mononuclear cells (PBMC) of COPD patients and in mice chronically exposed to cigarette smoke [18]. As reported in lung APC from these mice, we hypothesized that exposure to CSE might altered the response to *S. pneumoniae* in COPD patients and human DC.

To evaluate this, we first analyzed the pro-Th1 and -Th17 response to *S. pneumoniae* in PBMC from COPD patients. Since DC play a central role in the host response to bacteria, we evaluated the effects of cigarette smoke extract (CSE) on their capacity to initiate a Th17 response against *S. pneumoniae*, on bacteria uptake and on costimulatory molecule expression and cytokine secretion. We finally investigate the role of cytoplasmic and mitochondrial oxidative stress in CSE effects.

## Methods

### Cell preparation

Whole blood from anonymous healthy adult donors was obtained at the Etablissement Français du Sang (French National Blood Service). Peripheral blood mononuclear cells (PBMC) were isolated using Ficoll-paque gradient. Human monocytes were purified from PBMC by positive selection over a MACS column using anti-CD14-monoclonal antibodies conjugated microbeads (Miltenyi Biotec GmBH, Germany). Immature human monocyte-derived dendritic cells (MDDC) were generated by culturing monocytes for 5 days in RPMI 1640 supplemented with 10 % heat-inactivated fetal calf serum (FCS) (Invitrogen, Paisley, UK), IL-4 (10 ng/ml) and GM-CSF (25 ng/ml) (PromoCell, Heidelberg, Germany). Immature MDDC were characterized by their phenotype (CD11c^+^ CD1a^+^ HLA-DR^low^ CD83^low^ and CCR7^low^). Autologous T-cells were purified from whole blood by negative selection using the Pan T cell isolation kit II (Miltenyi Biotec GmBH) and stored at −80 °C in freezing mix (90 % FCS and 10 % DMSO). For the ex vivo study, whole blood was collected from 14 non-smokers healthy adults, 13 smokers without COPD and 9 stable COPD patients after informed consent (CPP 2008-A00690-55). Description of the patients is depicted in Table 1. PBMC were isolated as described above and stimulated as previously described [18].

**Table 1:** Clinical characteristics of COPD patients, smokers and non-smokers

Group	Nb	Sexe (M/F)	Age	Smoking (pack-year)	FEV1 %	PO_2_	BODE	Inhaled corticosteroid
COPD	9	8/1	57.8 ± 3.2	57 ± 5.9	57.8 ± 7.9	70.9 ± 2.3	2.4 ± 0.8	4
Smokers	13	10/3	42.6 ± 4.9	35.4 ± 4.6	93.6 ± 1.5	ND	ND	0
Non smokers	14	10/4	45.5 ± 5.7	0	95.3 ± 3.5	ND	ND	0

FEV1%, percentage of the forced expiratory lung volume in the first second; PO_2_, blood partial pressure of oxygen; BODE: index combining *Body mass index, airflow obstruction, dyspnea and exercise capacity (6-minute walk test)*. Results are expressed as mean ± SEM

*ND* not determined

### Preparation of cigarette smoke extract

Cigarette smoke extract (CSE) was prepared according to the method described by Blue and Janoff [19]. Briefly, the smoking apparatus consisted of a 60-ml syringe to which a cigarette was attached. CSE was prepared by drawing 60 ml of cigarette smoke through the filter into the syringe and then slowly bubbling the smoke into 10 ml of basal Airway Epithelial Cell Medium (PromoCell). Two Kentucky research cigarettes 3RF4 were smoked per 10 ml of medium. The final solution was filtered through 0.2 μm filters and used immediately at 4 % dilution.

### Streptococcus pneumoniae

Encapsulated *Streptococcus pneumoniae* serotype 1 (clinical isolate from University Hospital of Lille, France) was stored at −80 °C in 60 % glycerol. For infection, bacteria was expanded by re-suspension in Todd Hewitt broth supplemented with 2 % FCS and incubated at 37 °C for 4 h. The multiplicity of infection (MOI) used was 2 bacteria in exponential phase growth per 1 MDDC or PBMC.

### Activation of human MDDC and design of the coculture

Immature MDDC were exposed to 4 % CSE for 3 h in RPMI 1640 before activation by *S. pneumoniae* or by the Lipopolysaccharide as a positive control (LPS, *E.coli* serotype O55B5, 1 μg/ml) (Invivogen, San Diego, CA). To stop bacterial growth, 100 UI/ml Penicillin and 100 μg/ml Streptomycin were added to the culture medium 1 h after *S. pneumoniae* has been added. After an overnight incubation at 37 °C, supernatants were harvested and MDDC collected and divided in two groups, one to analyze their phenotype in flow cytometry and one to analyze their APC function in coculture with autologous T-cells (5 × 10^4^ MDDC per 5 × 10^5^ T-cells in 500 μL RPMI 1640 supplemented with 10 % FCS, 5 days at 37 °C). Cell viability assessed by trypan blue staining confirmed that exposure to CSE did not increase cell toxicity in unstimulated and *S. pneumoniae*-stimulated MDDC (*data not shown*). For cocultures, a condition with T-cells alone in a well coated by anti-CD3 antibodies (20 μg/ml, BD Biosciences, San Diego, CA) was used as a positive control of T-cells activation.

### Flow cytometry

MDDC were labelled (30 min at 4 °C) with different mix of FITC-conjugated anti-CD36, −CD86, −CD209, −CCR7 or -CD1a, PE-conjugated anti-B7H1, −CD80 or -CD54, APC-conjugated anti-CD83, −CD40 or -CD11c, PECy5-conjugated anti-HLA-DR and corresponding IgG isotype controls (BD Pharmingen™, BD Biosciences). After been washed and fixed in 0.25 % paraformaldehyde (PFA), MDDC were gated using FSC and SSC on a FACSCalibur flow cytometer with CellQuest software (BD biosciences). For intracellular staining, T-cells were incubated in RPMI 1640 supplemented with 10 % FCS plus brefeldin A (10 μg/ml) for 6 h at 37 °C. Cells were first labelled (30 min at 4 °C) with APC-Cy7-conjugated anti-CD45, Alexa Fluor 700-conjugated anti-CD4 and AmCyan-conjugated anti-CD8. After 15 min incubation, cells were fixed, permeabilized (Kit, BD Biosciences) and labelled with FITC-conjugated anti-IFN-γ, APC-conjugated anti-IL-17, PE-conjugated anti-IL-22 or the related isotype controls. Results are expressed as the difference between median fluorescence intensity (MFI) with the specific antibody and the isotype control (ΔMFI).

### Cytokines measurements

For In vitro and ex vivo studies, supernatants were collected 24 h after *S. pneumoniae* exposure, and stored at −20 °C. Concentrations of cytokines were determined by sandwich ELISA as described by the manufacturer for IFN-γ, IL-1β, IL-4, IL-6, CXCL8, IL-10, IL-17, IL-22, IL-23 (eBiosciences, San Diego, CA) and for TNF-α and IL-12p70 (R&D systems, Abingdon, UK).

### Real time quantitative PCR

Specific experiments were done to quantify the mRNA expression of markers for oxidative stress. After a 6-hours incubation with *S. pneumoniae*, MDDC were washed in PBS and homogenized and stored at −20 °C in TRIzol reagent (Invitrogen). RNA were extracted using successively chloroform, isopropyl alcohol and 75 % ethanol, and re-suspended into 30 μl RNase free water. Quantity and quality was determined by Nanodrop spectrophotometer using OD 260 nm for measuring concentration and 260/280 ratio for assessing the purity. Overall quality was also evaluated by electrophoresis through a 0.8 % agarose gel visualized using GelStar™ staining (Lonza, Rockland, USA). Total RNA was reverse transcribed using Superscript® III Platinum® Two-Step qRT-PCR Kit (Invitrogen, Paisley, Scotland). Real-Time PCR was performed in duplicates in 96-well plates using SYBR® Green Master mix (Invitrogen, Paisley, Scotland). Primer sequences are listed in Additional file 1. Relative mRNA quantities were calculated using the comparative Ct method normalized to human β-actin (2^-ΔΔCt^).

### Endocytosis and bactericidy of *S. pneumoniae* by MDDC

*S. pneumoniae* was first labelled with pHrodo™ SE (Molecular Probes®, Invitrogen®) and stored at 4 °C protected from light according to manufacturer’s instructions. CSE was added 3 h before MDDC dye-labelled *S. pneumoniae* exposure (MOI 20). After a 30 min-incubation at 37 °C, cells were washed and a flow cytometer analysis was immediately performed with 488 nm argon-ion laser using a R-phycoerythrin emission filter. One condition of MDDC was incubated at 4 °C as an endocytosis negative control. Results are expressed in MFI. Endocytosis of viable bacteria after a 3-hours CSE exposure (MOI 10), was studied in MDDC as described by Zhou [20].

### Oxidative stress and mitochondrial dysfunction assessment

Cellular and focused mitochondrial oxidative stress were quantified either after 3 and 6 h after *S. pneumoniae* exposure using H2DCFDA and MitoSOX™, respectively as described by the manufacturer (ThermoFisher). After 30 min incubation at 37 °C, cells were washed in PBS and analyzed in flow cytometry. For evaluation of oxidative stress involvement in CSE effects, the antioxidants N-Acetyl-Cystein (0.5 mM), tertiary butyl hydroquinone (5, 10 or 20 μM), butylated hydroxyanisole (20 μM) or MitoTEMPO (10 μM) were added to the MDDC medium 30 min before CSE exposure (Sigma, St Louis, MO). Finally, to test involvement of mitochondrial dysfunction, two inhibitors of mitochondrial electron transport chain inducing mitochondrial dysfunction and oxidative stress, rotenone (2 μM) and antimycin A (1 μM) (Sigma), were used separately instead of CSE.

### Statistical analysis

Results are expressed as mean ± S.E.M. The statistical significance of differences was calculated by a Wilcoxon rank-sum test when comparing two groups and a Friedman test with a post-hoc Wilcoxon test with Holm correction when comparing more than two groups (R version 3.2.3). *P*-value lower than 0.05 were considered as significant.

## Results

### PBMC from COPD patients exhibit a defective pro-Th1 and pro-Th17 response to *S. pneumoniae*

**Fig. 1:**
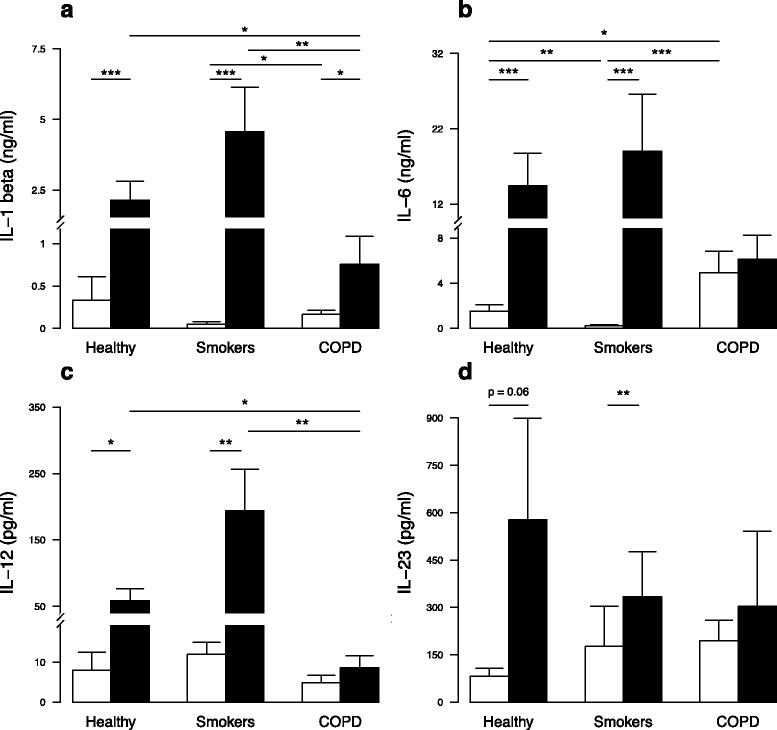
*S. pneumoniae*-induced cytokine secretion by PBMC from non-smoker healthy subjects (*n* = 14), smokers without COPD (*n* = 13) and COPD patients (*n* = 9). Supernatants were collected after 24 h incubation without stimulation (*white columns*) or after addition of *S. pneumoniae* (*black columns*). **a** IL-1β, **b** IL-6, **c** IL-12 and **d** IL-23 were quantified by ELISA. Data are reported as mean ± S.E.M. *P < 0.05, **P < 0.01, ***P < 0.001

To compare the cytokine profile of PBMC from healthy subjects, smokers and COPD patients, levels of CXCL8 (Additional file 2), IL-1β, IL-6, IL-12 and IL-23 were evaluated (Fig. 1). At baseline, PBMC from COPD patients produced more IL-6, but not IL-12 and IL-23, than healthy controls and smokers and more CXCL8 and IL-1β than smokers. As expected, *S. pneumoniae* exposure triggered higher secretion by PBMC from healthy controls of CXCL8, IL-1β, IL-6, and IL-12 with a same trend for IL-23. Interestingly, the same pattern was observed with PBMC from smokers. However, *S. pneumoniae* exposure did not induce IL-6, IL-12 and IL-23 secretion by PBMC from COPD patients whereas it increased IL-1β (Fig. 1). However, the concentrations of IL-1β in *S. pneumoniae*-stimulated PBMC were lower in COPD patients than in smokers. CXCL8 production by PBMC from COPD patients activated by *S. pneumoniae* was unchanged although the CXCL8 levels were similar in the 3 groups (Additional file 2). These data suggest that the response to *S. pneumoniae* is altered in PBMC from COPD patients but not those from smokers.

### CSE inhibits *S. pneumoniae*-induced MDDC maturation

**Fig. 2:**
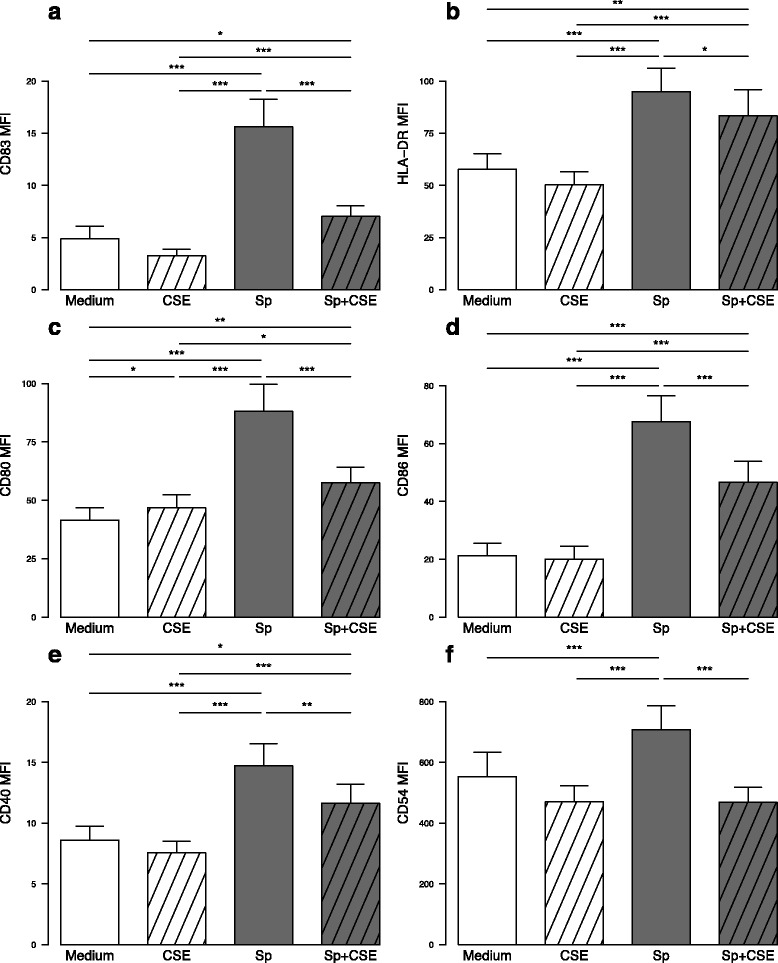
In vitro exposure to cigarette smoke extract (CSE) modulate the phenotype of monocyte-derived dendritic cells (MDDC) from healthy subjects activated by *S. pneumoniae* (Sp). Expression of **a** CD83, **b** HLA-DR, **c** CD80, **d** CD86, **e** CD40 and **f** CD54 was evaluated by flow cytometry in MDDC exposed to CSE and then activated or not by *S. pneumoniae* for 24 h. Data are reported as mean fluorescence intensity (MFI) ± S.E.M. of 27 experiments. *P < 0.05, **P < 0.01, ***P < 0.001

**Fig. 3:**
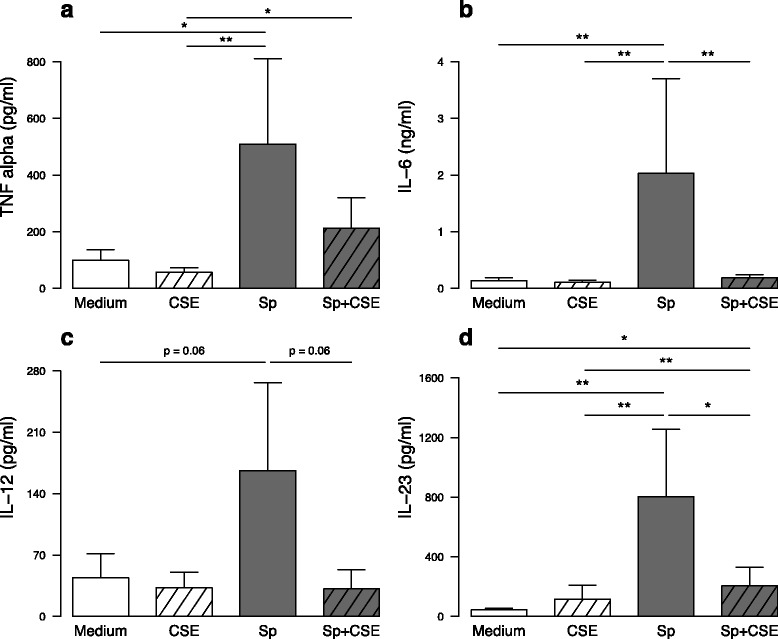
In vitro exposure to cigarette smoke extract (CSE) modulate the secretion of cytokines by monocyte-derived dendritic cells (MDDC) from healthy subjects activated by *S. pneumoniae* (Sp). Levels of **a** TNF-α, **b** IL-6, **c** IL-12 and **d** IL-23 were quantified by ELISA in MDDC culture supernatants collected after 24 h incubation with CSE and/or *S. pneumoniae*. Data are reported as mean ± S.E.M. of 14 experiments. *P < 0.05, **P < 0.01

Since we observed a defective production of cytokines that are known to be mainly produced by APC, in PBMC from COPD patients, we next focused on the effect of CSE on DC. Stimulation with *S. pneumoniae* significantly increased the expression of maturation markers including CD83, HLA-DR, CD80, CD86, CD40 and CD54 on MDDC (Fig. 2). In contrast, pre-exposure of MDDC with CSE modulated the phenotypic response to *S. pneumoniae* by minimizing the expression of all these molecules. As a note, CSE itself did not have any impact on MDDC phenotype except a small increase of CD80 expression. Addition of *S. pneumoniae* also increased the secretion of pro-inflammatory cytokines including TNF-α, IL-6, IL-23 and CXCL8 with a similar trend for IL-12 by MDDC (Fig. 3 and Additional file 3a). CSE exposure inhibited IL-6 and IL-23 secretion in *S. pneumoniae*-activated MDDC with a similar trend for TNF-α and IL-12 (Fig. 3). CSE alone only modulated the CXCL8 levels for higher levels (Additional file 3a-b). As a control, CSE was also partly able to inhibit the LPS-response by MDDC by inhibiting CD83, CD40 and CD54 expression and TNF-α, IL-6, IL-12 and IL-23 secretion (Additional files 3 and 4). All of these data showed that CSE inhibits *S. pneumoniae*-induced MDDC maturation and secretion of cytokines involved in Th1 and Th17 T-cell differentiation.

### CSE-exposed MDDC are responsible for a defective Th1 and Th17 response to *S. pneumoniae*

**Fig. 4:**
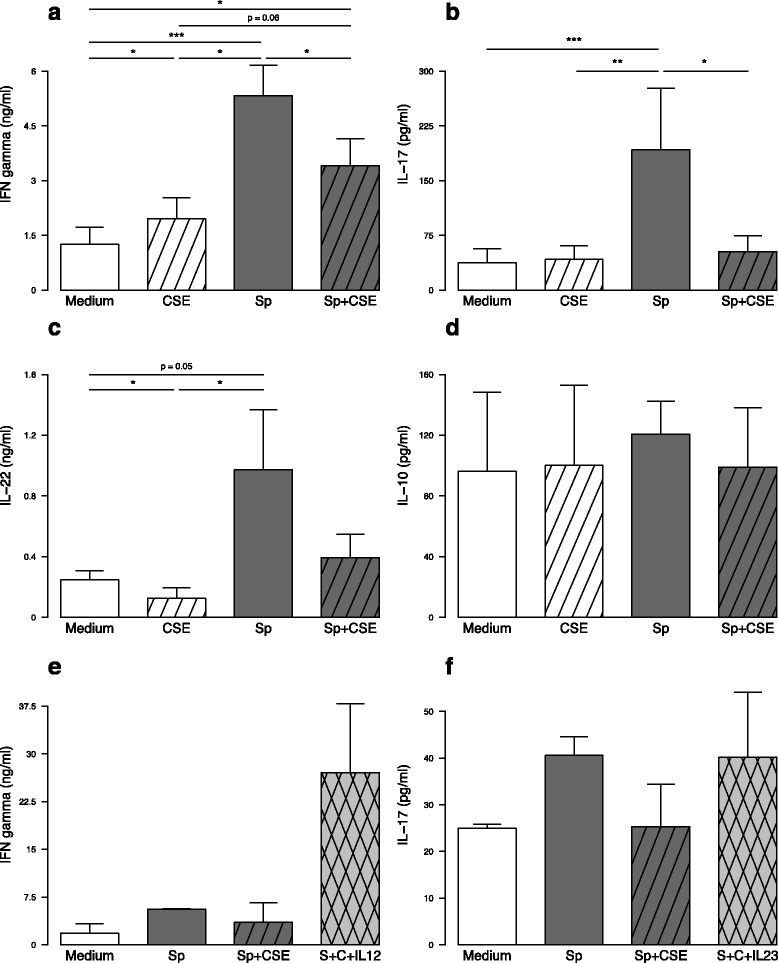
In vitro exposure to cigarette smoke extract (CSE) of monocyte-derived dendritic cells (MDDC) altered their ability to prime T-cells. MDDC were exposed to CSE and then activated or not by *S. pneumoniae* (Sp). After 24 h incubation, MDDC were then cocultured 5 days with autologous T-cells. Levels of **a** IFN-γ, **b** IL-17, **c** IL-22 and **d** IL-10 were quantified by ELISA in coculture supernatants. In some experiments, either recombinant human IL-12 or IL-23 were added to the cocultures with CSE- and Sp-exposed MDDC in order to quantify their ability to restore **e** IFN-γ or **f** IL-17 secretion, respectively. Data are reported as mean ± S.E.M. of 14 and 3 experiments for a-d and e-f, respectively. *P < 0.05, **P < 0.01, ***P < 0.001

To evaluate the effects of CSE on the crosstalk MDDC/T-cells, we next performed coculture experiments between MDDC and autologous T-cells. T-cells cultured with *S. pneumoniae*-exposed MDDC secreted significantly higher levels of IFN-γ, IL-17 and IL-22 (Fig. 4a−c). Intracellular staining confirmed the T-cell origin of these cytokines (Additional file 5). In contrast, when MDDC were pre-exposed to CSE prior *S. pneumoniae* activation, T-cells secreted lower levels of IFN-γ and IL-17 with a similar trend for IL-22 (Fig. 4a−c). MDDC only exposed to CSE had no effect on IL-17 secretion but induced a slightly higher IFN-γ and lower IL-22 production (*p* < 0.05). Exposure to CSE had no impact on IL-10 and IL-4 levels (Fig. 4d and *data not shown*).

To demonstrate that the defective production of IL-12 and IL-23 by MDDC following CSE exposure was responsible for the defective IFN-γ and IL-17 production by T-cells respectively, we supplemented the cocultures with these recombinant cytokines. As depicted in Fig. 4e and f, addition of recombinant human IL-12 and IL-23 to cocultures induced a huge IFN-γ secretion and restored IL-17 secretion, respectively. Although, the difference was not significant due to the small sample size, the effect is reproducible. These data showed that inhibition of MDDC maturation by CSE altered their ability to promote Th1 and Th17 T-cell differentiation, an effect that seems to be related to the decreased secretion of IL-12 and IL-23 by MDDC.

### Impact of CSE on *S. pneumoniae* endocytosis by MDDC

**Fig. 5:**
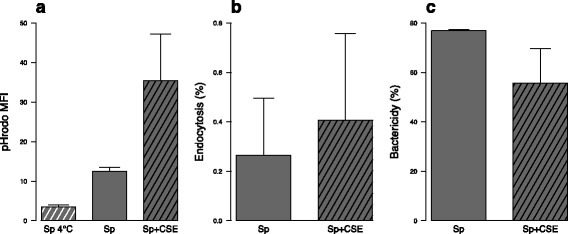
In vitro exposure to cigarette smoke extract (CSE) is associated with higher endocytosis of *S. pneumoniae* (Sp) by monocyte-derived dendritic cells (MDDC). **a** Mean fluorescence intensity (MFI) of internalized dye-labelled Sp was measured by flow cytometry. One condition was put at 4 °C to inhibit endocytosis as a negative control. **b** Proportion of viable internalized Sp after one hour exposure were quantify to measure bacteria endocytosis as described by Zhou [20]. **c** After one more hour incubation, internalized still viable Sp were quantify to measure the proportion of killed Sp. Data represent mean ± S.E.M. of 4 and 2 experiments for a and b-c respectively. There was no statistical difference between groups

To understand why CSE limits MDDC maturation induced by *S. pneumoniae*, bacterial endocytosis was first investigated. Despite CSE inhibition of *S. pneumoniae*- and LPS-induced MDDC expression of the adherence molecule CD54 (Fig. 2f and Additional file 4f), MDDC exposure to dye-labeled *S. pneumoniae* showed higher fluorescence in CSE pre-exposed cells (Fig. 5a). As pHrodo™ is only fluorescent in acidic environment, it indirectly reflected a greater internalization of bacteria. This result was comforted with viable bacteria showing a trend to a higher endocytosis and a decreased killing of internalized bacteria in MDDC pre-exposed to CSE (Fig. 5b−c). These results showed that CSE inhibition of MDDC maturations is not related to decreased bacterial endocytosis.

### CSE induced oxidative stress was not implicated in the altered response to *S. Pneumoniae*

**Fig. 6:**
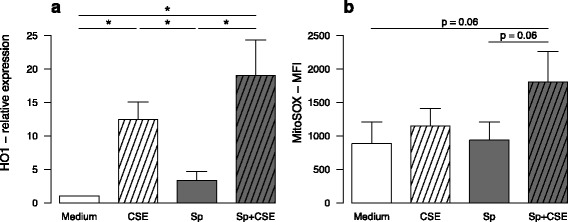
In vitro exposure to cigarette smoke extract (CSE) induced an oxidative stress in monocyte-derived dendritic cell (MDDC) from healthy subjects activated by *S. pneumoniae* (Sp). **a** Expression of Heme-oxygenase (HO)-1 was evaluated by qRT-PCR in MDDC exposed to CSE and then activated or not by *S. pneumoniae* for 6 h. **b** Mitochondrial oxidative stress was measured by flow cytometry by measuring the fluorescence of MitoSox in MDDC exposed to CSE and then activated or not by *S. pneumoniae* for 6 h. Data represent mean ± S.E.M of 4 and 6 experiments for a and b, respectively. *P < 0.05

**Fig. 7:**
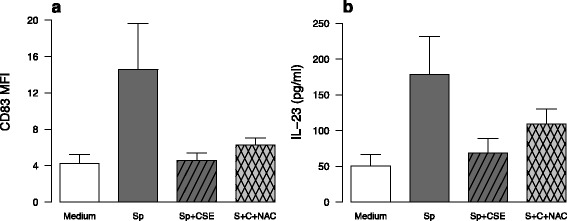
Treatment with the anti-oxidant N-acetylcystein (NAC) did not reverse the inhibitory effect of cigarette smoke extract (CSE) on the phenotype and the secretion of cytokines by monocyte-derived dendritic cell (MDDC) activated by *S. pneumoniae* (Sp). The expression of (**a**) CD83 and the secretion of (**b**) IL-23 by MDDC treated or not with NAC and then exposed to CSE and Sp for 24 h were evaluated by flow cytometry and ELISA, respectively. Data are reported as mean ± S.E.M of 6 experiments. There was no statistical difference between groups

**Fig. 8:**
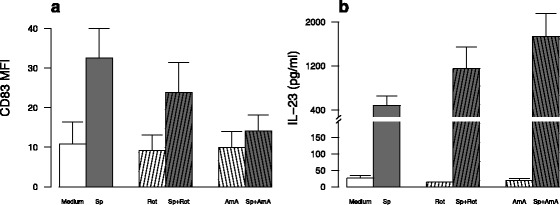
In vitro exposures to rotenone (Rot) and antimycin A (AmA) inhibit the phenotype of monocyte-derived dendritic cells (MDDC) activated by *S. pneumoniae* (Sp) but not the secretion of cytokines. **a** Expression of CD83 and **b** the secretion of IL-23 were evaluated by flow cytometry and ELISA, respectively, in MDDC exposed to inhibitors and then activated or not by *S. pneumoniae* for 24 h. Data represent mean ± S.E.M of 6 and 4 experiments for **a** and **b**, respectively. There was no statistical difference between groups

We next focused on the impact of oxidative stress in the repressive effect of CSE on DC maturation. As expected, CSE exposure, despite the presence or not of *S. pneumoniae*, increased the expression of heme oxygenase 1 (HO1) within MDDC (Fig. 6a). This was confirmed by increased fluorescence in DCFDA-pre-treated MDDC after 3 h incubation (*data not shown*). Oxidative stress involvement in CSE inhibitory effects was next tested by adding before CSE exposure, antioxidants targeting different oxidative pathways. After addition of N-Acetyl-Cystein (NAC), the inhibitory effect of CSE on co-maturation marker expression and cytokine expression was not reversed except for CD80 and CD86 for which NAC tended to increase their expression (*p* = NS) (Fig. 7, Additional file 6). Similarly, two other antioxidants, tertiary butyl hydroquinone (TBHQ) and butylated hydroxyanisole (BHA) were not able to reverse CSE inhibitory effects (*data not shown*). Whereas cytoplasmic oxidative stress did not persist after 6 h incubation, co-activation with CSE and *S. pneumoniae* induced a mitochondrial oxidative stress depicted by increased MitoSOX fluorescence (Fig. 6b). Again, MitoTEMPO a mitochondrially-targeted antioxidant could not reverse CSE inhibitory effects (*data not shown*). Moreover, MDDC pre-treatment by NAC and MitoTEMPO did not restore the production of IFN-γ and IL-17 by T-cells in cocultures (*data not shown*). Furthermore, treatment with rotenone and antimycin A, which induced a mitochondrial dysfunction, tended to minimize *S. pneumoniae*-induced MDDC CD83 expression (Fig. 8a) as well as *S. pneumoniae*-stimulated maturation marker expression (Additional file 7). In contrast, cytokine production by *S. pneumoniae*-activated MDDC was not modified by addition of rotenone and antimycin A as illustrated by sustained *S. pneumoniae*-induced IL-23 secretion (Fig. 8b). These data suggest that CSE-induced mitochondrial stress does not reproduce the major effects of CSE.

## Discussion

In this study, we observed that PBMC from COPD patients secrete lower pro-Th1 and -Th17 cytokines in response to *S. pneumoniae* strengthening the concept of an APC defective response to pathogens in this disease. We demonstrated that in vitro exposure to CSE inhibits MDDC maturation and induction of specific Th1 and Th17 responses against *S. pneumoniae*. In contrast to previous studies using LPS [14], anti-oxidants did not markedly reverse these effects suggesting that cigarette smoke (CS) inhibitory effects are not mainly related to oxidative stress in the context of infection with a live bacteria.

Previous studies have reported higher pro-inflammatory cytokines blood levels in COPD patients at steady state compared to controls [21]. Besides, others studies have also showed increased pro-inflammatory cytokines levels during exacerbations compared to steady state [22]. Whereas in our study, the levels of IL-1β, IL-6 and CXCL8 were higher in unstimulated cells from COPD patients, we report for the first time that PBMC from COPD patients exhibit a defective pro-inflammatory cytokine (including IL-1β, IL-6, IL-12 and IL-23) secretion in response to bacteria compared to controls or to smokers. This defective response may favor airway bacterial infection and colonization which is a main feature of the disease [23]. This is comforted by another study reporting lower IL-17 blood levels in COPD patients with opportunistic pathogen colonization [17]. These pro-inflammatory cytokines are mainly produced by APC and are involved in induction of Th1 and Th17 responses to pathogens. Therefore, these results strengthen our previous data showing a defective Th17 response to *S. pneumoniae* in PBMC from COPD patients and in mice chronically exposed to CS. This defect in mice is related to an altered activation of alveolar macrophages and DC by *S. pneumoniae* and suggest that the altered Th17 response to pathogens in COPD patients might be due to a defective response of both DC and macrophages [18].

DC are professional APC linking innate and adaptive immune responses which play a central role in the COPD immunopathology [8]. There are discrepant data on DC features in the lung of COPD patients either reporting an accumulation of DC or a decreased number of matured DC in small airways of COPD patients [24, 25]. This might be related to the different clinical status or potentially to the presence of dysbiosis and/or colonization in the airways of these patients. A recent clinical study based on lung biopsy showed decreased CD83 expression in DC from small airways of COPD patients as well as smokers compared with healthy non smokers, corroborating cigarette smoke involvement in DC decreased maturation even in subjects without COPD [26]. Surprisingly, in our study, the cytokine secretion in response to *S. pneumoniae* in smokers was not altered compared to non smokers suggesting that this defective response may be a specific feature of the disease. In our model of mice chronically exposed to cigarette smoke, we report a decreased lung DC maturation and a lower production of IL-1β and IL-23 in response to *S. pneumoniae* suggesting that both in human and mice, the defect in these cytokines explained the altered Th17 cytokine production induced by CS [18]. Therefore, all these data as well as ours suggest that cigarette smoke exposure locally inhibits lung APC maturation even in smokers without COPD. In contrast, defective systemic response to bacteria is a specific feature of COPD which may be the consequence of the conjugate effect of exposure to CS, systemic chronic inflammation and/or host predisposition to develop the disease.

After demonstrating the inhibitory effects of CSE on DC maturation, we would like to decipher those mechanisms. In a recent study, Givi et al. has reported that short-term CSE exposure induced maturation of DC whereas 10 days exposure suppress it [27]. In our model, both short-term and 6 days exposure (*data not shown*) had inhibitory effects on DC maturation. We think that these differences may be linked to the use of different cells, namely murine bone marrow derived DC and Hodgkin’s disease-derived cell line L428, and of cigarette smoke from commercial cigarette without filter. In fact, other authors using human MDDC have reported that exposure to CSE of LPS-activated DC decreased IL-12 secretion and expression of costimulatory molecules [13, 14]. Moreover, they showed that secretion of IL-12 can be partially restored by treatment with the antioxidants N-acetylcysteine and catalase. Conversely, in our model we were not able to restore DC maturation and cytokine secretion with physiologic concentrations of N-acetylcysteine nor with two other antioxidants, TBHQ and BHA. Interestingly, we observed that exposure to CSE induced a mitochondrial oxidative stress. As previously reported, inducing a mitochondrial dysfunction by blocking mitochondrial electron transport chain with rotenone or antimycin A inhibits expression of maturation associated molecules [28]. However, it could not reproduce all CSE inhibition as these molecules had no effects on cytokines secretion. Moreover, the mitochondria-targeted antioxidant did not reversed the inhibitory effect of CSE in bacteria-activated DC. These data indicate that CSE-induced oxidative stress is not essential for the inhibitory effect on response to bacteria. Recent reports demonstrated that exposure to cigarette smoke also modulated miRNA expression [29]. In our model, we observed in preliminary experiments that exposure to CSE inhibits the expression of miR22, a miRNA involved in the development of COPD through its effect on DC and the synthesis of IL-17 [30]. However, in our hands, the inhibition of miR22 as well as its upregulation with a mimicker did not allow to reproduce the phenotype of CSE-exposed MDDC. Altogether, we can suspect that since the effect of CSE, a very complex atmosphere, involved different targets, the modulation of one of them did not allow to block its major effects.

Bacteria endocytosis was higher in CSE pre-exposed DC. This contrasts with previous data on alveolar macrophages showing decreased *S. pneumoniae* internalization after concentrated ambient particles exposure [20]. However, Phipps et al. have shown that CS exposure of alveolar macrophages reduced complement-mediated endocytosis of *S. pneumoniae* while unopsonized bacteria endocytosis was sustained [31]. This suggests that CS exposure may affect only the complement-dependent endocytosis pathway and not the others. As endocytic activity is inversely correlated to the degree of maturation in DC [32], this indicates that the inhibition by CSE of maturation might explain the sustained bacteria endocytosis. Although this effect should be confirmed, we observed decreased intracellular killing of *S. pneumoniae* in CSE pre-treated DC suggesting that bacteria may persist inside DC promoting bacterial colonization and subsequent re-infection. Other extracellular bacteria like *Streptococcus pyogenes* or intracellular like *Coxiella burnetii* and *Brucella abortus* have demonstrated ability to persist inside macrophages or DC respectively by inhibiting phagolysosome trafficking [33, 34]. Interestingly, these effects are associated with decreased DC maturation. Furthermore, Tardif et al. have demonstrated that heme-oxygenase-1 which was increased in our model and carbon monoxide which is an important component of cigarette smoke could both block endosome-lysosome fusion without endocytosis reduction in LPS-exposed DC [35]. Altogether these data suggest that the inhibitory effects of CSE may be related to defective phagolysosome trafficking.

## Conclusions

In this study, we observed a defective pro-Th1 and -Th17 response to *S. pneumoniae* in the PBMC of COPD patients compared to healthy controls and smokers suggesting an altered response to bacteria in APC during COPD. Moreover, we showed that this may be explained by cigarette smoke inhibition of DC capacity to activate antigen specific T-cell response, an effect which may implicate an altered phagolysosome trafficking. Further studies are needed to confirm this hypothesis. Nevertheless, these data suggest that new therapeutics targeting this defect may be helpful to improve treatment and prevention of COPD exacerbations.

## Abbreviations

APC, antigen-presenting cell; BHA, butylated hydroxyanisole; COPD, chronic obstructive pulmonary disease; CS, cigarette smoke; CSE, cigarette smoke extract; DC, dendritic cells; DCFDA, dichlorofluorescin diacetate; FCS, fetal calf serum; IL, interleukin; LPS, lipopolysaccharide; MDDC, monocyte-derived dendritic cells; MOI, multiplicity of infection; PBMC, peripheral blood mononuclear cells; TBHQ, tertiary butyl hydroquinone

## Additional files

Additional file 1: Primer sequences. (DOCX 35 kb) Additional file 2:
*S.pneumoniae*-induced CXCL8 secretion by PBMC from non-smoker healthy subjects (*n* = 14), smokers without COPD (*n* = 13) and COPD patients (*n* = 9). Supernatants were collected after 24 h incubation without stimulation (white columns) or after addition of *S.pneumoniae* (black column). Data are reported as mean ± S.E.M. **P* < 0.05, ***P* <0.01. (PDF 29 kb) Additional file 3: In vitro exposure to cigarette smoke extract (CSE) modulate the secretion of cytokines by monocyte-derived dendritic cells (MDDC) from healthy subjects activated by *S.pneumoniae* (Sp) (a) or by LPS (b-f). Levels of CXCL8 (a-b), TNF alpha (c), IL-6 (d), IL-12 (e) and IL-23 (f) were quantified by ELISA in MDDC culture supernatants collected after 24 h incubation with CSE and Sp or LPS. Data are reported as mean ± S.E.M. of 12 experiments. **P* < 0.05, ***P* < 0.01, ****P* < 0.001. (PDF 46 kb) Additional file 4: In vitro exposure to cigarette smoke extract (CSE) modulate the phenotype of monocyte-derived dendritic cells (MDDC) from healthy subjects activated by LPS. Expression of CD83 (a), HLA-DR (b), CD80 (c), CD86 (d), CD40 (e) and CD54 (f) was evaluated by flow cytometry in MDDC exposed to CSE and then activated or not by LPS for 24 h. Data are reported as mean fluorescence intensity (MFI) ± S.E.M. of 20 experiments. **P* < 0.05, ***P* < 0.01, ****P* < 0.001. (PDF 44 kb) Additional file 5: T-cells intracellular staining strategy of gaiting. Cells were first gated using foward scatter (FSC) and side scatter (SSC) and next, T-cells were separated on CD8^+^ and CD4^+^ based on fluorescence of these two markers on CD45^+^ cells. IL-17 ans IFN-γ positive cells were calculated in comparison with baseline fluorescence of isotype controls. (PDF 422 kb) Additional file 6: Treatment with the anti-oxidant N-acetylcystein (NAC) did not reverse the inhibitory effect of cigarette smoke extract (CSE) on the phenotype of monocyte-derived dendritic cells (MDDC) activated by *S.pneumoniae* (Sp). The expression of HLA-DR (a), CD80 (b), CD86 (c), CD40 (d) and CD54 (e) by MDDC treated or not with NAC and then exposed to CSE and Sp for 24 h were evaluated by flow cytometry. Data are reported as mean ± S.E.M. of 6 experiments. (PDF 39 kb) Additional file 7: In vitro exposure to rotenone (Rot) or antimycin A (AmA) inhibits the phenotype of monocyte-derived dendritic cells (MDDC) activated by *S.pneumoniae* (Sp). Expression of HLA-DR (a), CD80 (b), CD86 (c), CD40 (d) and CD54 (e) were evaluated by flow cytometry in MDDC exposed to rotenone or antimycin A and then activated or not by *S.pneumoniae* for 24 h. Data represent mean ± S.E.M. of 6 experitments. There were no statistical differences between groups. (PDF 50 kb)
