# Supplementary material for: Cigarette smoke alters the ability of human dendritic cells to promote anti-Streptococcus pneumoniae Th17 response
Source: Respir Res. 2016 Jul 26;17:94. doi: 10.1186/s12931-016-0408-6 (PMC4962368; doi:10.1186/s12931-016-0408-6)
Supplement: Additional file 1: — Primer sequences. (DOCX 35 kb) [file 12931_2016_408_MOESM1_ESM.docx]

**Additional File 1**

**Primer sequences**

| **Primers** | **Forward** | **Reverse** |
| --- | --- | --- |
| β-actin | 5’-TCCTCACCCTGAAGTACCCCA-3’ | 5’-AGCCACACGCAGCTCATTGT-3’ |
| HO1 | 5’-ATGGCCTCCCTGTACCACATC-3’ | 5’-TGTTGCGCTCAATCTCCTCCT-3’ |
