# Supplementary material for: Cigarette smoke alters the ability of human dendritic cells to promote anti-Streptococcus pneumoniae Th17 response
Source: Respir Res. 2016 Jul 26;17:94. doi: 10.1186/s12931-016-0408-6 (PMC4962368; doi:10.1186/s12931-016-0408-6)
Supplement: Additional file 2: — S.pneumoniae-induced CXCL8 secretion by PBMC from non-smoker healthy subjects (n = 14), smokers without COPD (n = 13) and COPD patients (n = 9). Supernatants were collected after 24 h incubation without stimulation (white columns) or after addition of S.pneumoniae (black column). Data are reported as mean ± S.E.M. *P < 0.05, **P <0.01. (PDF 29 kb) [file 12931_2016_408_MOESM2_ESM.pdf]

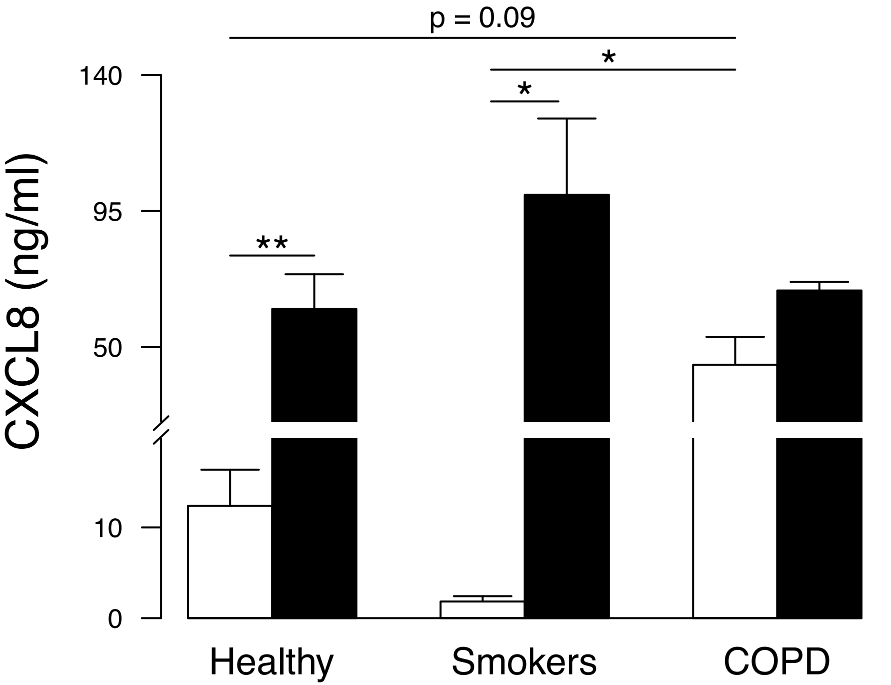

Additional file 2: S.pneumoniae-induced CXCL8 secretion by PBMC from non-smoker healthy subjects (n=14), smokers without COPD (n=13) and COPD patients (n=9). Supernatants were collected after 24h incubation without stimulation (white columns) or after addition of S.pneumoniae (black column). Data are reported as mean +/- S.E.M. * $P<0.05$, ** $P<0.01$.
