# Supplementary material for: Cigarette smoke alters the ability of human dendritic cells to promote anti-Streptococcus pneumoniae Th17 response
Source: Respir Res. 2016 Jul 26;17:94. doi: 10.1186/s12931-016-0408-6 (PMC4962368; doi:10.1186/s12931-016-0408-6)
Supplement: Additional file 3: — In vitro exposure to cigarette smoke extract (CSE) modulate the secretion of cytokines by monocyte-derived dendritic cells (MDDC) from healthy subjects activated by S.pneumoniae (Sp) (a) or by LPS (b-f). Levels of CXCL8 (a-b), TNF alpha (c), IL-6 (d), IL-12 (e) and IL-23 (f) were quantified by ELISA in MDDC culture supernatants collected after 24 h incubation with CSE and Sp or LPS. Data are reported as mean ± S.E.M. of 12 experiments. *P < 0.05, **P < 0.01, ***P < 0.001. (PDF 46 kb) [file 12931_2016_408_MOESM3_ESM.pdf]

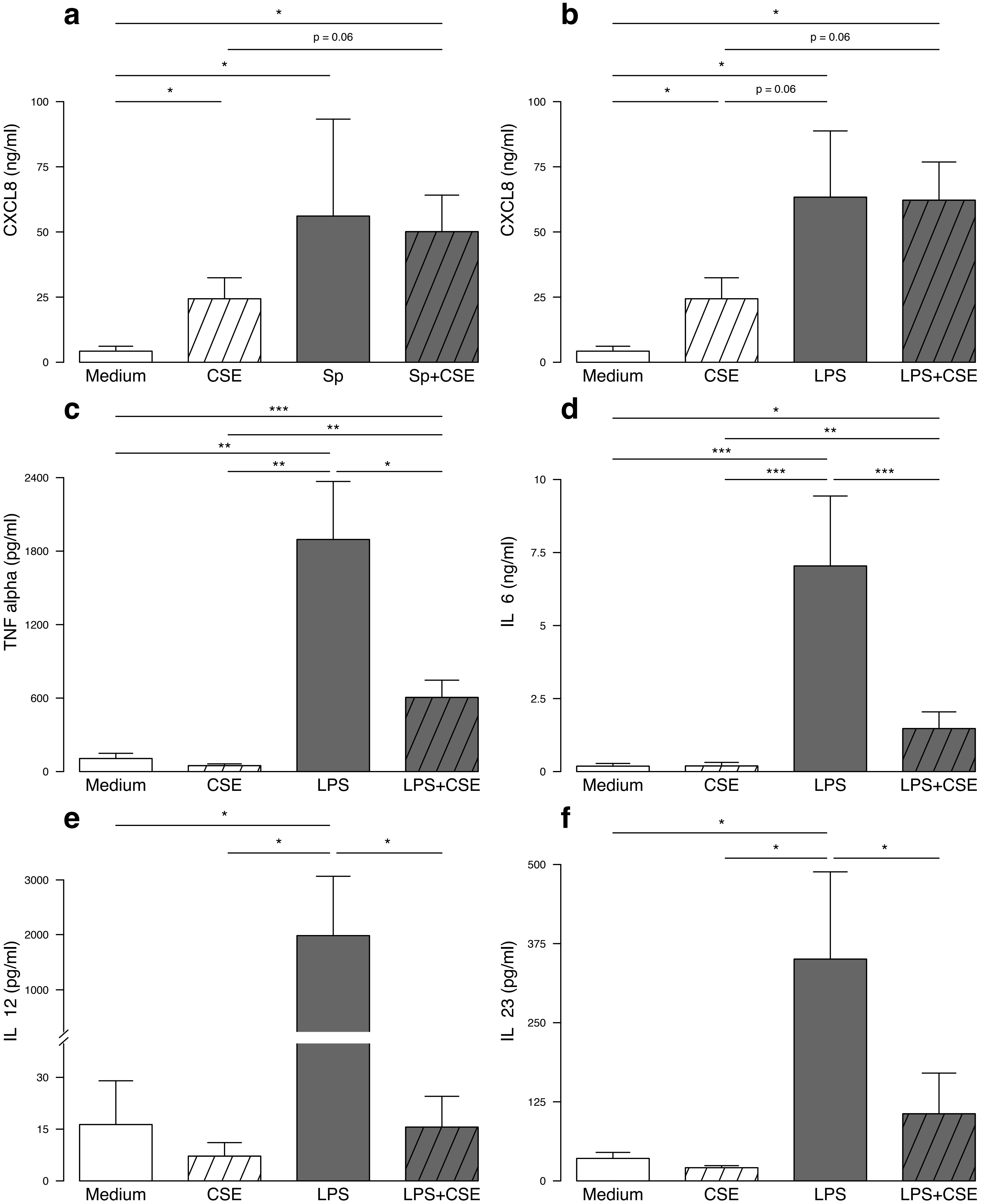

Additional file 3: In vitro exposure to cigarette smoke extract (CSE) modulate the secretion of cytokines by monocyte-derived dendritic cells (MDDC) from healthy subjects activated by (a) S. pneumoniae (Sp) or by (b-f) LPS. Levels of (a-b) CXCL8, (c) TNF alpha, (d) IL-6, (e) IL-12 and (f) IL-23 were quantified by ELISA in MDDC culture supernatants collected after 24 hours incubation with CSE and Sp or LPS. Data are reported as mean +/- S.E.M. of 12 experiments. *P<0.05, **P<0.01, ***P<0.001.
