# Supplementary material for: Cigarette smoke alters the ability of human dendritic cells to promote anti-Streptococcus pneumoniae Th17 response
Source: Respir Res. 2016 Jul 26;17:94. doi: 10.1186/s12931-016-0408-6 (PMC4962368; doi:10.1186/s12931-016-0408-6)
Supplement: Additional file 4: — In vitro exposure to cigarette smoke extract (CSE) modulate the phenotype of monocyte-derived dendritic cells (MDDC) from healthy subjects activated by LPS. Expression of CD83 (a), HLA-DR (b), CD80 (c), CD86 (d), CD40 (e) and CD54 (f) was evaluated by flow cytometry in MDDC exposed to CSE and then activated or not by LPS for 24 h. Data are reported as mean fluorescence intensity (MFI) ± S.E.M. of 20 experiments. *P < 0.05, **P < 0.01, ***P < 0.001. (PDF 44 kb) [file 12931_2016_408_MOESM4_ESM.pdf]

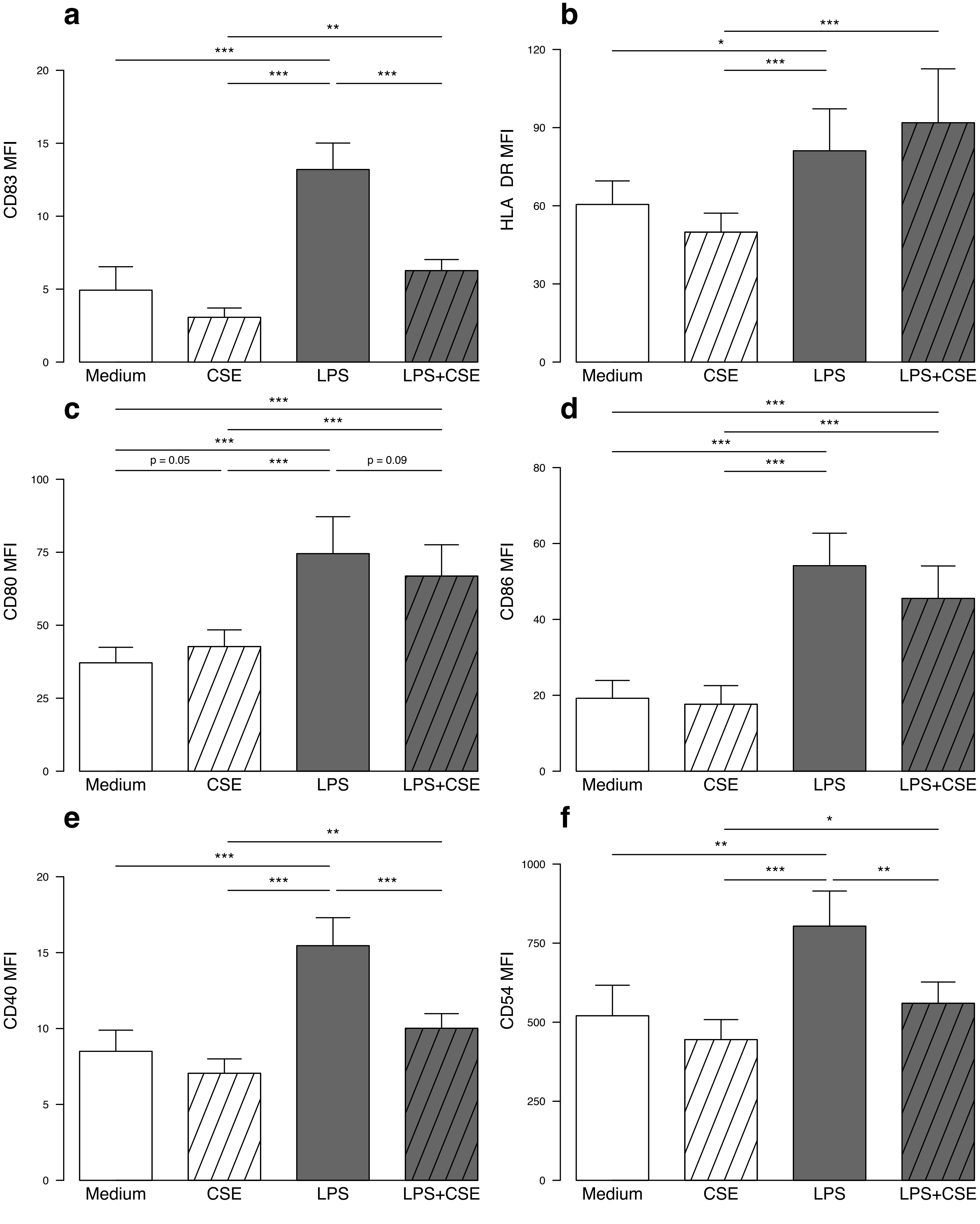

Additional file 4: In vitro exposure to cigarette smoke extract (CSE) modulate the phenotype of monocyte-derived dendritic cells (MDDC) from healthy subjects activated by LPS. Expression of (a) CD83, (b) HLA-DR, (c) CD80, (d) CD86, (e) CD40 and (f) CD54 was evaluated byflow cytometry in MDDC exposed to CSE and then activated or not by LPS for 24 hours. Data are reported as mean fluorescence intensity (MFI) +/- S.E.M. of 20 experiments. *$P<0.05$, **$P<0.01$, ***$P<0.001$.
