# Supplementary figures and images for: Cigarette smoke alters the ability of human dendritic cells to promote anti-Streptococcus pneumoniae Th17 response
Source: Respir Res. 2016 Jul 26;17:94. doi: 10.1186/s12931-016-0408-6 (PMC4962368; doi:10.1186/s12931-016-0408-6)

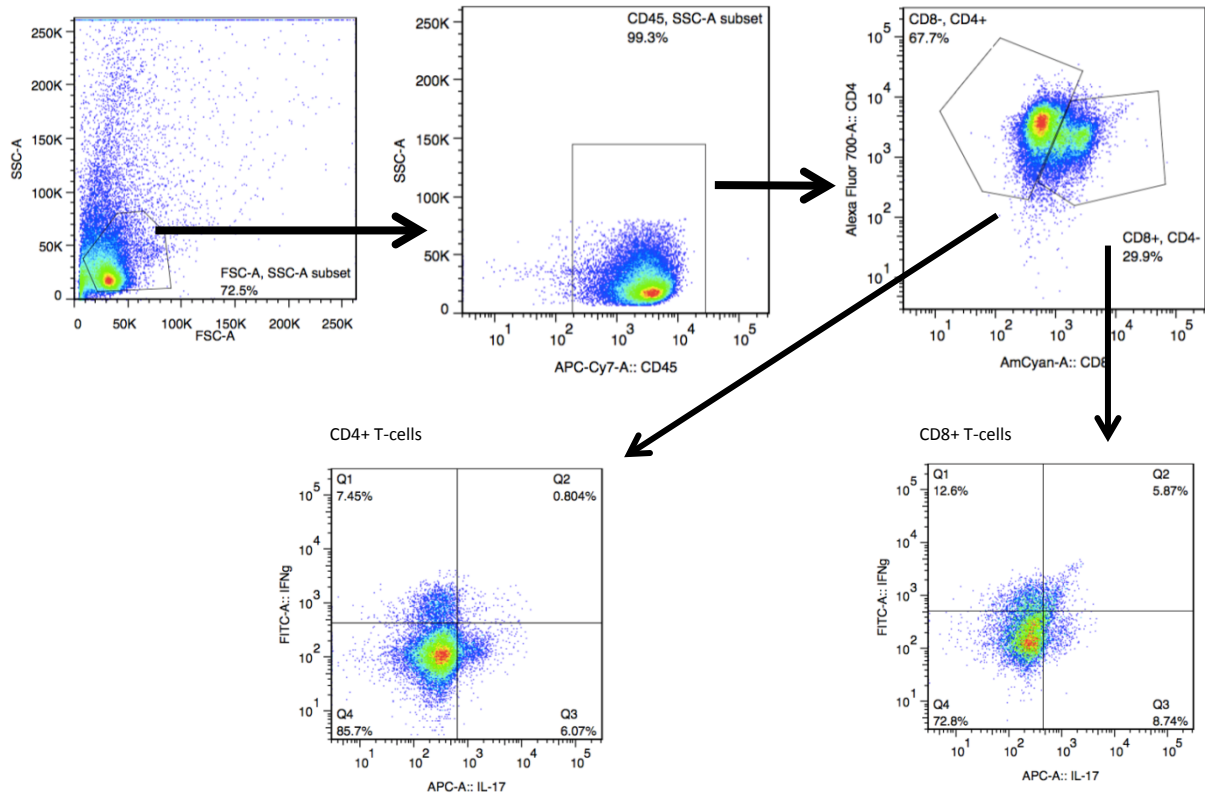

Supplement: Additional file 5: — T-cells intracellular staining strategy of gaiting. Cells were first gated using foward scatter (FSC) and side scatter (SSC) and next, T-cells were separated on CD8+ and CD4+ based on fluorescence of these two markers on CD45+ cells. IL-17 ans IFN-γ positive cells were calculated in comparison with baseline fluorescence of isotype controls. (PDF 422 kb) [file 12931_2016_408_MOESM5_ESM.pdf]
