# Supplementary material for: Cigarette smoke alters the ability of human dendritic cells to promote anti-Streptococcus pneumoniae Th17 response
Source: Respir Res. 2016 Jul 26;17:94. doi: 10.1186/s12931-016-0408-6 (PMC4962368; doi:10.1186/s12931-016-0408-6)
Supplement: Additional file 6: — Treatment with the anti-oxidant N-acetylcystein (NAC) did not reverse the inhibitory effect of cigarette smoke extract (CSE) on the phenotype of monocyte-derived dendritic cells (MDDC) activated by S.pneumoniae (Sp). The expression of HLA-DR (a), CD80 (b), CD86 (c), CD40 (d) and CD54 (e) by MDDC treated or not with NAC and then exposed to CSE and Sp for 24 h were evaluated by flow cytometry. Data are reported as mean ± S.E.M. of 6 experiments. (PDF 39 kb) [file 12931_2016_408_MOESM6_ESM.pdf]

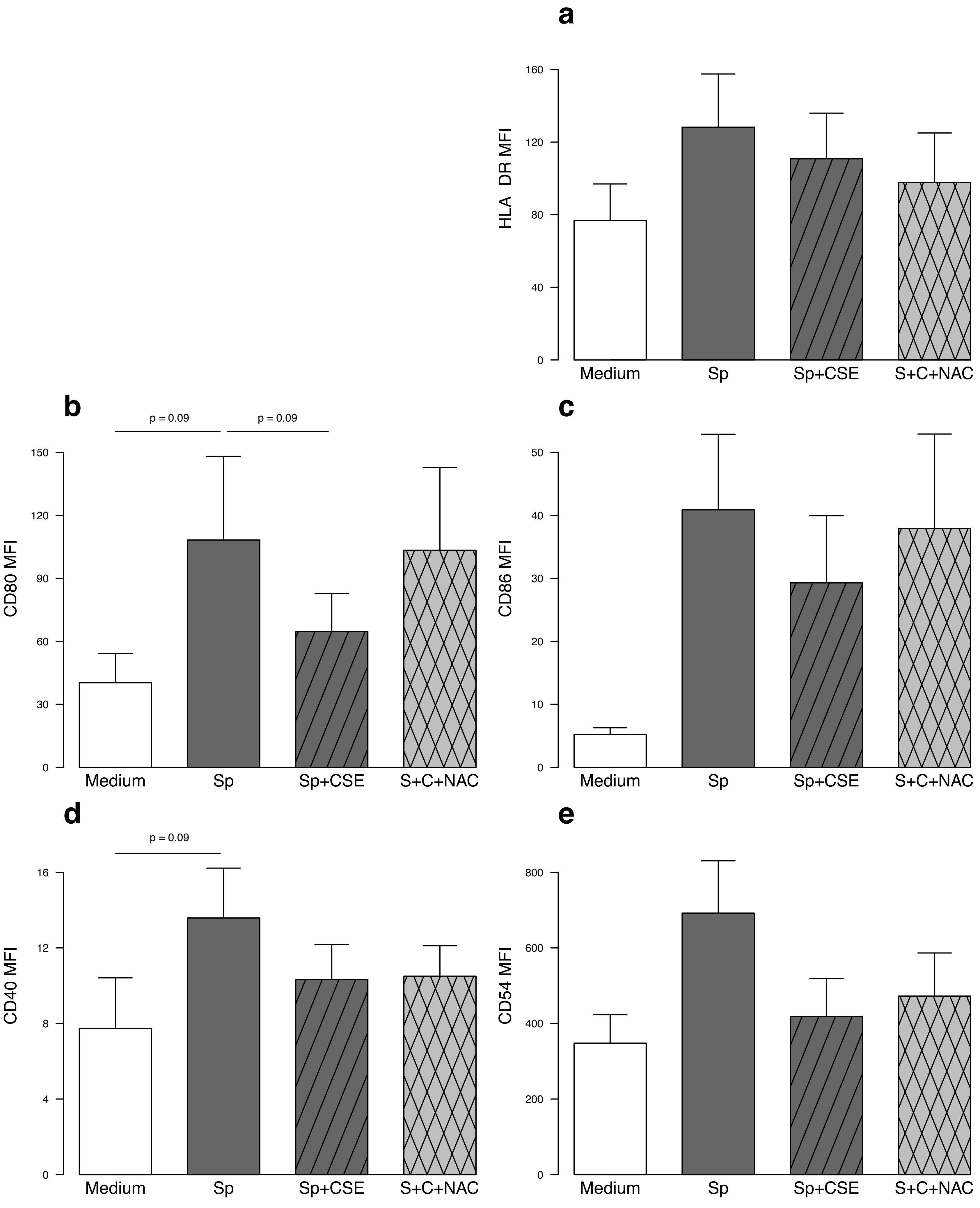

Additional file 6: Treatment with the anti-oxidant N-acetylcystein (NAC) did not reverse the inhibitory effect of cigarette smoke extract (CSE) on the phenotype of monocyte-derived dendritic cells (MDDC) activated by S. pneumoniae (Sp). The expression of (a) HLA-DR, (b) CD80, (c) CD86, (d) CD40 and (e) CD54by MDDC treated or not with NAC and then exposed to CSE and Sp for 24 hours were evaluated by flow cytometry. Data are reported as mean +/- S.E.M. of 6 experiments
