# Supplementary material for: Cigarette smoke alters the ability of human dendritic cells to promote anti-Streptococcus pneumoniae Th17 response
Source: Respir Res. 2016 Jul 26;17:94. doi: 10.1186/s12931-016-0408-6 (PMC4962368; doi:10.1186/s12931-016-0408-6)
Supplement: Additional file 7: — In vitro exposure to rotenone (Rot) or antimycin A (AmA) inhibits the phenotype of monocyte-derived dendritic cells (MDDC) activated by S.pneumoniae (Sp). Expression of HLA-DR (a), CD80 (b), CD86 (c), CD40 (d) and CD54 (e) were evaluated by flow cytometry in MDDC exposed to rotenone or antimycin A and then activated or not by S.pneumoniae for 24 h. Data represent mean ± S.E.M. of 6 experitments. There were no statistical differences between groups. (PDF 50 kb) [file 12931_2016_408_MOESM7_ESM.pdf]

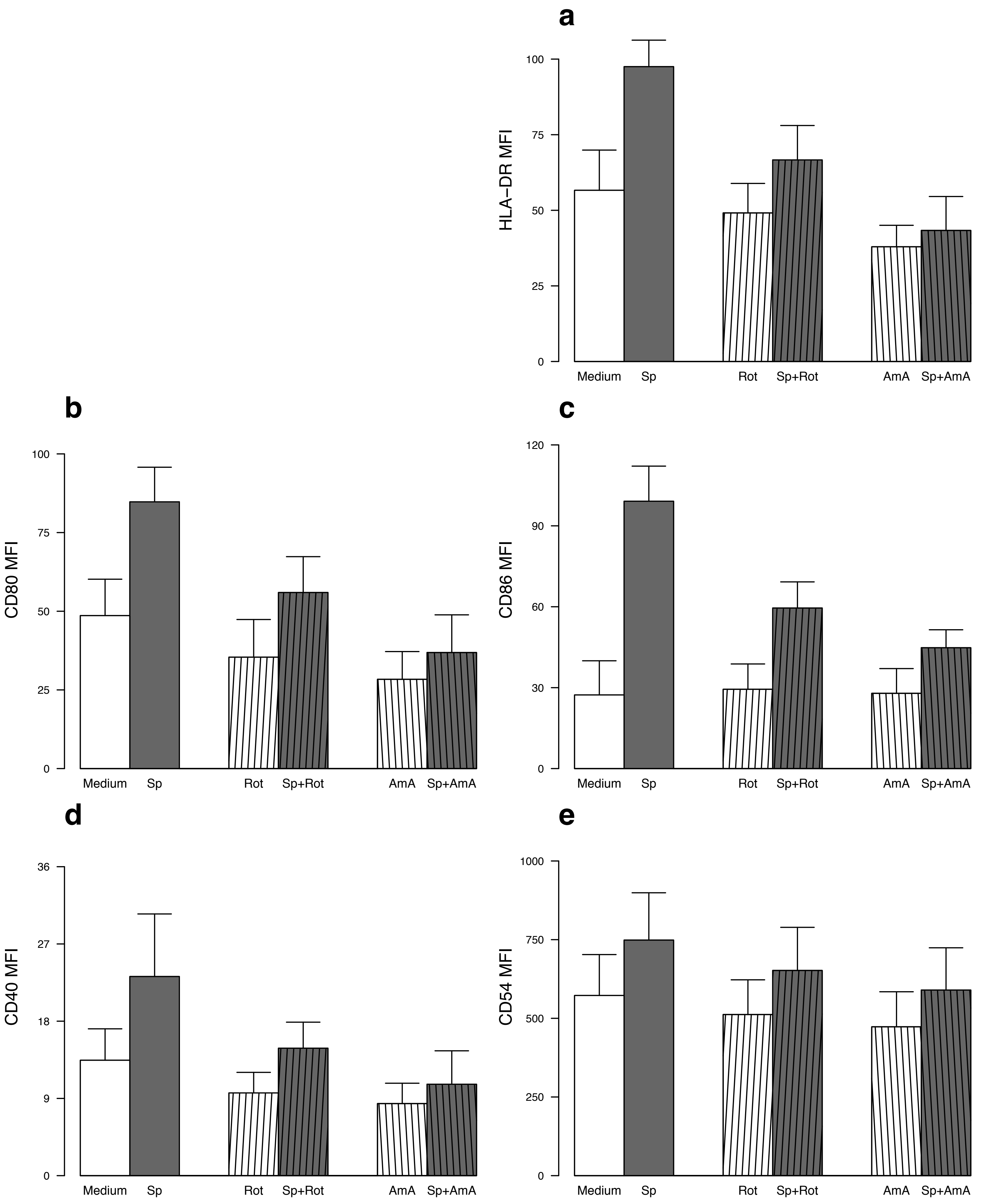

Additional file 7: In vitro exposure to rotenone (Rot) or antimycin A (AmA) inhibits the phenotype of monocyte-derived denritic cells (MDDC) activated by S. pneumoniae (Sp). Expression of (a) HLA-DR, (b) CD80, (c) CD86, (d) CD40 and (e) CD54 were evaluated by flow cytometry in MDDC exposed to rotenone or antimycin A and then activated or not by S. pneumoniae for 24 hours. Data represent mean +/- S.E.M. of 6 experiments. There were no statistical differences between groups.
